# Loss of β-Cytoplasmic Actin in the Intestinal Epithelium Increases Gut Barrier Permeability *in vivo* and Exaggerates the Severity of Experimental Colitis

**DOI:** 10.3389/fcell.2020.588836

**Published:** 2020-10-23

**Authors:** Susana Lechuga, Nayden G. Naydenov, Alex Feygin, Michael Cruise, James M. Ervasti, Andrei I. Ivanov

**Affiliations:** ^1^Department of Inflammation and Immunity, Lerner Research Institute, Cleveland Clinic Foundation, Cleveland, OH, United States; ^2^School of Nursing, Virginia Commonwealth University School of Nursing, Richmond, VA, United States; ^3^Department of Pathology, Cleveland Clinic Foundation, Cleveland, OH, United States; ^4^Department of Biochemistry and Molecular Biology, University of Minnesota Medical School, Minneapolis, MN, United States

**Keywords:** actin isoforms, cytoskeleton, barrier, tight junctions, adherens junctions, mucosal inflammation, colitis, cells death

## Abstract

Intestinal epithelial barrier is critical for the maintenance of normal gut homeostasis and disruption of this barrier may trigger or exaggerate mucosal inflammation. The actin cytoskeleton is a key regulator of barrier structure and function, controlling the assembly and permeability of epithelial adherens and tight junctions. Epithelial cells express two actin isoforms: a β-cytoplasmic actin and γ-cytoplasmic actin. Our previous *in vitro* studies demonstrated that these actin isoforms play distinctive roles in establishing the intestinal epithelial barrier, by controlling the organization of different junctional complexes. It remains unknown, whether β-actin and γ-actin have unique or redundant functions in regulating the gut barrier *in vivo*. To address this question, we selectively knocked out β-actin expression in mouse intestinal epithelium. Mice with intestinal epithelial knockout of β-actin do not display gastrointestinal abnormalities or gross alterations of colonic mucosal architecture. This could be due to compensatory upregulation of γ-actin expression. Despite such compensation, β-actin knockout mice demonstrate increased intestinal permeability. Furthermore, these animals show more severe clinical symptoms during dextran sodium sulfate induced colitis, compared to control littermates. Such exaggerated colitis is associated with the higher expression of inflammatory cytokines, increased macrophage infiltration in the gut, and accelerated mucosal cell death. Consistently, intestinal organoids generated from β-actin knockout mice are more sensitive to tumor necrosis factor induced cell death, *ex vivo*. Overall, our data suggests that β-actin functions as an essential regulator of gut barrier integrity *in vivo*, and plays a tissue protective role during mucosal injury and inflammation.

## Introduction

The actin cytoskeleton is a key regulator of epithelial homeostasis. Assembly of prominent actin filament bundles is required for the formation of diverse cellular structures, such as intercellular junctions and apical microvilli in differentiated epithelial cell monolayers, cell matrix adhesions in migrating cells, and the cleavage furrow that separates dividing cells (Ivanov, 2008; Crawley et al., 2014; Braga, 2016; Dekraker et al., 2018; Rothenberg and Fernandez-Gonzalez, 2019). Additionally, a dynamic network of actin filaments is associated with cytoplasmic organelles and nuclear transcriptional complexes (Plessner and Grosse, 2019; Ravichandran et al., 2020). The actin cytoskeleton participates in virtually all housekeeping and specialized epithelial functions. Importantly, it could control these functions at different levels: from setting up the nuclear transcriptional rheostat for cell stemness and differentiation, to regulating the stability and dynamics of effector structures and the cell cortex (Braga, 2016; Misu et al., 2017; Viita and Vartiainen, 2017; Dekraker et al., 2018; Rothenberg and Fernandez-Gonzalez, 2019).

The actin cytoskeleton is formed by self-association of the most abundant cellular protein, actin (Dominguez and Holmes, 2011; Pollard, 2016). Actin reversibly polymerizes into polar filaments, and this process is strictly controlled by different actin-binding proteins (Dominguez, 2009; Barr-Gillespie, 2015; Pollard, 2016; Buracco et al., 2019). Mammals have six actin genes encoding different actin isoforms (Vandekerckhove and Weber, 1978). Two of them, β-cytoplasmic actin and γ-cytoplasmic actin (referred to thereafter as β-actin and γ-actin), are expressed in epithelial cells (Perrin and Ervasti, 2010; Kashina, 2020). β-actin and γ-actin possess remarkable structural similarity, being different only in 4 amino acid residues at the N-terminal part of the molecule (Perrin and Ervasti, 2010). In spite of this similarity, β-actin and γ-actin could play unique roles in cultured fibroblasts, epithelial, and cancer cells by participating in different molecular events during cell proliferation, differentiation, and motility (Bunnell et al., 2011; Lechuga et al., 2014; Dugina et al., 2015, 2018; Patrinostro et al., 2017; Malek et al., 2020). Additionally, studies in mouse models with tissue specific depletion of either β-actin, or γ-actin have demonstrated both the unique and redundant functions of these actin isoforms in the regulation of myogenesis, auditory cell function, brain development, and synaptic transmission *in vivo* (Sonnemann et al., 2006; Perrin et al., 2010; Cheever et al., 2012; Wu et al., 2016; Madsen et al., 2018).

Through the control of the structure and stability of epithelial tight junctions (TJ) and adherens junctions (AJ), the actin cytoskeleton is a well-recognized regulator of epithelial barriers (Ivanov, 2008; Ivanov et al., 2010; Mege and Ishiyama, 2017; Sluysmans et al., 2017). Epithelial junctions are formed at the plasma membrane via multiple interactions between different transmembrane and cytosolic plaque proteins (Troyanovsky, 2012; Suzuki, 2013; Takeichi, 2014; Van Itallie and Anderson, 2014). Transmembrane TJ and AJ proteins, such as claudins, occludin, junctional adhesion molecule-A (JAM-A), E-cadherin, and nectins, directly engage in homotypic adhesions to their partners on the opposing epithelial plasma membrane (Troyanovsky, 2012; Suzuki, 2013; Takeichi, 2014; Van Itallie and Anderson, 2014). Cytosolic plaque constituents, including *zonula occludens* (ZO) proteins, α-catenin, β-catenin and p120-catenin, enhance the adhesive properties of transmembrane TJ/AJ proteins and couple them to different intracellular structures (Troyanovsky, 2012; Suzuki, 2013; Takeichi, 2014; Van Itallie and Anderson, 2014).

Both AJ and TJ directly associate with the elaborate and dynamic cortical actin cytoskeleton (Ivanov, 2008; Braga, 2016). The perijunctional actin filaments generate the mechanical forces that control all stages of junctional biogenesis, including AJ/TJ assembly, maintenance, and disassembly (Ivanov, 2008; Mege and Ishiyama, 2017; Sluysmans et al., 2017; Charras and Yap, 2018; Varadarajan et al., 2019). An essential role of the actin cytoskeleton in controlling junctional integrity and function has been demonstrated with studies that utilized actin filament depolymerizing drugs to trigger robust TJ and AJ disassembly and epithelial barrier disruption (Madara et al., 1986; Vasioukhin et al., 2000; Ivanov et al., 2005; Shen and Turner, 2005). In model epithelial cell monolayers, both β-actin and γ-actin participate in the formation of the perijunctional actin cytoskeleton, though these actin isoforms appear to be selectively associated with different junctional complexes (Baranwal et al., 2012). For example, in intestinal epithelial cell monolayers γ-actin predominantly incorporates into stable TJ-associated F-actin bundles, whereas the more mobile β-actin-based filaments primarily affiliate with AJ (Baranwal et al., 2012). Remarkably, depletion of either β-actin, or γ-actin causes selective disruption of AJ and TJ structure, respectively, and leads to an increase in paracellular permeability (Baranwal et al., 2012). A fraction of β-actin mRNA is accumulated and locally translated at AJ in renal epithelial and myoblast cells (Rodriguez et al., 2006; Gutierrez et al., 2014). Inhibition of such local perijunctional synthesis of β-actin attenuates AJ assembly (Gutierrez et al., 2014; Cruz et al., 2015). The described studies suggest that β-actin and γ-actin cooperate during the establishment of model epithelial barriers *in vitro* by controlling the assembly and stability of different junctional complexes. However, it remains unclear if similar functional interplay between these two actin isoforms is essential for epithelial barrier integrity *in vivo*. Importantly, elucidating the actin isoform-dependent regulation of epithelial barriers could lend significant insight into understanding the pathogenesis of human immune disorders, including inflammatory bowel diseases (IBD). One of the key manifestations of IBD is increased permeability of the gut barrier, which is readily recapitulated in animal models of experimental colitis (Marchiando et al., 2010; Lee et al., 2018; Fasano, 2020; Schlegel et al., 2020). Interestingly, published proteomic studies of the intestinal mucosa of IBD patients and animal models of colitis report a marked dysregulation in the expression of β-actin and γ-actin in the inflamed gut (Shkoda et al., 2007; Cooney et al., 2016; Moriggi et al., 2017). It is possible that alterations to actin isoform levels could destabilize the intestinal epithelial barrier, thereby contributing to the development of mucosal inflammation. To gain insights into the actin-dependent regulation of the gut barrier in normal and inflamed intestinal mucosa, we generated and characterized a mouse model with intestinal epithelial-specific knockout of β-actin. Our data suggests that intestinal epithelial β-actin acts as an essential regulator of mucosal barrier integrity in healthy gut, and limits mucosal injury and inflammation during experimental colitis *in vivo.*

## Materials and Methods

### Antibodies and Other Reagents

Primary antibodies that were used to detect cytoskeletal, junctional, and leukocyte proteins by immunofluorescence labeling and immunoblotting analysis are listed in a Supplementary Table 1. Alexa Fluor-488-conjugated donkey anti-rabbit, donkey anti-mouse and donkey anti-goat, Alexa Fluor-555-conjugated donkey anti-mouse and goat anti-rat secondary antibodies, and Alexa Fluor-488-labeled phalloidin were obtained from Thermo Fisher Scientific (Waltham, MA). Horseradish peroxidase-conjugated goat anti-rabbit and anti-mouse secondary antibodies were acquired from Bio-Rad Laboratories (Hercules, CA). All other chemicals were obtained from Thermo Fisher Scientific, or Millipore-Sigma (Saint Louis, MO).

### Animals

In order to establish a conditional knockout of β-actin in the intestinal epithelium, Actb ^*flox/flox*^ mice on a C57BL/6 background (Sonnemann et al., 2006; Perrin et al., 2010) were crossed with villin-Cre animals (Jackson Laboratory, stock # 004586). In these villin-Cre mice, a 12.4 kb fragment of mouse villin 1 promoter directs Cre recombinase expression in both the small intestine and the colon (Madison et al., 2002). The animal colony was maintained under pathogen-free conditions in the vivarium of Virginia Commonwealth University Medical Center and then Lerner Research Institute of Cleveland Clinic. The mouse room was on a 12 h light/dark cycle and standard feed and tap water were available, *ad libitum*. At the beginning of colitis experiments, mice weighed 18–25 g, with no significant difference between the body masses of mice of different genotypes. All procedures were conducted under animal research protocols approved by the Virginia Commonwealth University and Lerner Research Institute Animal Care and Use Committees in accordance with the National Institutes of Health Animal Care and Use Guidelines.

### Induction and Characterization of Dextran Sulfate-Induced Colitis

Experimental colitis was induced in 8–10 week old β-actin cKO mice by administering a 3% (w/v) solution of dextran sulfate, sodium salt (DSS, Thermo Fisher Scientific), in drinking water, *ad libitum*. Either Actb ^*flox/flox*^ or villin-Cre only littermates were used as controls. Vehicle-treated animals received tap water. Both male and female mice were used at roughly equal numbers. Animals were weighed and monitored for symptoms of gastrointestinal disorder daily. The disease activity index was calculated as previously described, by averaging numerical scores of body weight loss, stool consistency, and intestinal bleeding (Rhee et al., 2012). With regards to body weight, no weight loss was scored as 0, loss of 1–5% was scored as 1; 5–10% and 10–15% weight loss was scored as 2 and 3, respectively, whereas more than 15% weight loss was scored as 4. For stool consistency, well-formed pellet was scored as 0, soft and semi-formed stool as 2, and liquid stool or diarrhea was scored as 4. For intestinal bleeding, no blood was scored as 0, hemoccult-positive stool as 2, and gross rectal bleeding was scored as 4. On day 7 of DSS administration, animals were euthanized, with their colonic tissue harvested and separated into several segments. The samples were either fixed in 10% formaldehyde solution, snap frozen in liquid nitrogen, or embedded into an optimal cutting temperature medium and snap frozen for subsequent histological and biochemical examination. Formalin-fixed samples were paraffin embedded, sectioned, and stained with hematoxylin and eosin (H&E). The H&E stained slides were examined, “blind,” by a gastrointestinal pathologist, and the tissue injury index was calculated as previously described (D’Haens et al., 1998; Mahler et al., 1998; Ding et al., 2012). The index represents the sum of individual scores reflecting mucosal inflammation, leukocyte infiltration, fibrosis and epithelial erosion. Briefly, inflammation was graded as follows: 0, no evidence of inflammation; 1, low level of inflammation with scattered infiltrating cells; 2, moderate inflammation with multiple sites of infiltration; 3, high level of inflammation with increased vascular density and marked wall thickening; and 4, severe inflammation with transmural leukocyte inflammation with loss of goblet cells. Fibrosis was graded as follows: 1, no evidence of fibrosis (collagen covering < 5% of area), 2, loose irregular connective tissue (6–15%); 3, increase in number and density of focal collagen (16–40%); and 4, severe presence of thick collagen layer (collagen covering more than 40% of the area). Ulceration score was graded as 0, no presence; 1, yes presence, less than 25%; 2, yes, 25–50%; 3, yes, greater than 50%; and 4, yes, into *muscularis propria*. Apoptosis score was graded as follows: 0, none; 1, mild superficial; 2, mild base of crypt; 3, confluent apoptosis; and 4, glandular loss from apoptosis.

### Measurement of Intestinal Epithelial Barrier Permeability *in vivo*

*In vivo* intestinal permeability assay was performed in β-actin cKO and control animals receiving either 3% DSS or water for 7 days. Water and food was withdrawn for 3 h before the start of the procedure. Animals were gavaged with fluorescein isothiocyanate (FITC)-labeled dextran (4,000 Da; 60 mg/100 g body weight) and euthanized 3 h later for blood collection via cardiac puncture. Blood serum was obtained by centrifugation, and FITC fluorescence intensity was measured using a Victor3 V plate reader (Perkin Elmer, Waltham, MA) with excitation and emission wavelengths at 485 and 544 nm, respectively. The measured value of FITC-dextran-free serum was subtracted from each individual measurement. The concentration of FITC-dextran in blood serum was calculated using SigmaPlot v12.5 software, based on a plotted standard curve prepared via serial dilutions of a 60 mg/ml stock solution of FITC-dextran in phosphate buffered saline (PBS).

### Immunoblotting Analysis

Mouse colonic segments were harvested, longitudinally bisected, and washed with ice-cold PBS. Epithelial cells were collected by gently scraping the exposed interior with a razor blade, and snap frozen in liquid nitrogen for further analysis. Intestinal epithelial scrapings were lysed and homogenized in a RIPA buffer containing a protease inhibitor cocktail and phosphatase inhibitor cocktails 2 and 3 (Millipore-Sigma). Samples were diluted with 2x SDS sample loading buffer and boiled. SDS-polyacrylamide gel electrophoresis was conducted using standard protocols with an equal amount of total protein loaded per lane (10 or 20 μg), followed by immunoblotting on nitrocellulose membrane. Protein expression was quantified via densitometry using Image J software (National Institutes of Health, Bethesda MD).

### Quantitative Real-Time RT-PCR

Total RNA was isolated from whole colonic segments of β-actin cKO and control animals using a mirVana miRNA Isolation kit (Thermo Fisher Scientific) followed by DNase treatment to remove genomic DNA. Total RNA (1 μg) was reverse transcribed using an iScript cDNA synthesis kit (Bio-Rad Laboratories). Quantitative real-time RT-PCR was performed using iTaq Universal SYBR Green Supermix (Bio-Rad Laboratories) in a 7900HT Fast Real-time PCR System (Applied Biosystems, Foster City, CA). The primer sequences have been published in our previous study (Naydenov et al., 2016). The threshold cycle number (Ct) for specific genes of interest and a housekeeping gene were determined based on the amplification curve representing a plot of the fluorescent signal intensity vs. the cycle number. The relative expression of each gene was calculated by a comparative Ct method that is based on the inverse proportionality between Ct and the initial template concentration (2^–ΔΔ^ ^*Ct*^), as previously described (Ivanov et al., 2002). This method is based on two-step calculations of ΔCt = Ct_*targetgene*_ − Ct_*GAPDH*_ and ΔΔCt = ΔCt_*e*_−ΔCt_*c*_, where index e refers to the sample from any DSS or water-treated β-actin cKO, or control mice, and index c refers to the sample from a water-treated control animal assigned as an internal control.

### Immunofluorescence Labeling, Cell Death Assay, and Confocal Microscopy

Full thickness frozen colonic sections were fixed with 4% paraformaldehyde and permeabilized with absolute methanol to label for pSTAT and leukocyte markers. Paraformaldehyde-fixed and Triton-X100-permeabilized frozen sections were utilized for F-actin labeling. Formalin fixed and paraffin embedded full thickness colonic sections were used to immunolabel junctional proteins and actin isoforms. Following standard deparaffinization and antigen retrieval, sections were blocked for 60 min in Hanks HEPES-buffered salt solution containing 1% bovine serum albumin, followed by overnight incubation at 4°C with primary antibodies. Samples were then washed and incubated with Alexa dye-conjugated secondary antibodies for 60 min, and finally rinsed with blocking buffer. F-actin was visualized after 60 min labeling with Alexa-555-labeled phalloidin. Vector TrueView reagents mix (Vector Laboratories, Burlingame, CA) was applied to quench tissue autofluorescence. Labeled samples were mounted on slides using ProLong Antifade mounting reagent with or without DAPI (Thermo Fisher Scientific). Cell death in colonic tissue sections was evaluated with a terminal deoxynucleotidyl transferase dUTP nick end labeling (TUNEL) assay, using an ApopTag Fluorescein *in situ* Apoptosis Detection Kit (Millipore-Sigma), according to the manufacturer’s instructions. Fluorescently labeled tissue sections were imaged using a Leica SP8 confocal microscope (Wentzler, Germany). The Alexa Fluor 488 and 555 signals were acquired sequentially in frame-interlace mode, to eliminate cross talk between channels. Images were processed using Adobe Photoshop.

To quantify CD4, F4/80, MPO, and pSTAT3 immunolabeling, signal intensities were measured at the colonic surface and in the crypt areas. For TUNEL assay, individual dead cells were counted. Mean values were calculated by averaging the signal intensities obtained from the tissue samples of 5–7 different animals from each experimental group. The animal numbers for each experimental group are presented in figure legends.

### Culture of Intestinal Enteroids

Enteroids were generated from isolated small intestinal crypts of β-actin cKO and control mice, as previously described (Lechuga et al., 2017). Briefly, mice were euthanized and their small intestinal segments were dissected, longitudinally opened, and washed with ice-cold PBS. Crypts were released using 30 min incubation with PBS containing 2 mM of EDTA at 4°C, with constant agitation, followed by mechanical shaking. Debris and villous fragments were discarded, and the resulting crypt fraction was collected by centrifugation and resuspended in growth factor reduced Matrigel (BD Bioscience). After Matrigel polymerization, DMEM/F12 medium containing HEPES, glutamine, N2 and B27 supplements, and growth factors [50 ng/ml epidermal growth factor, 500 ng/ml R-spondin 1, and 100 ng/ml Noggin (R&D Systems)] were added. Intestinal enteroids were allowed to differentiate for 7 days and were observed using a bright field microscope (Olympus BX41, Japan). Cell death was induced by treating enteroids with 100 ng/ml of murine tumor necrosis factor (TNF)-α (PeproTech, Cranbury, NJ) for 12 h. Viable and dead enteroids were distinguished by morphology, using a bright-field microscope (Keyence, Osaka, Japan) and counted. At least 50 enteroids per experimental group were examined. The percentage of dead enteroids was calculated from 3 independent experiments.

### Statistical Analysis

Data are given as a mean ± SEM. The statistical significance of the difference between 2 sets of data was evaluated using the two tailed unpaired Student’s *t*-test when data were distributed normally. Differences in body weight loss and diseases activity index data were examined for statistical significance using one-way ANOVA (SigmaPlot 12.5 package). Statistical significance was accepted at *p* < 0.05.

## Results

### Intestinal Epithelial Specific Knockout of β-Actin Increases the Permeability of Normal Gut Barrier *in vivo*

**FIGURE 1:**
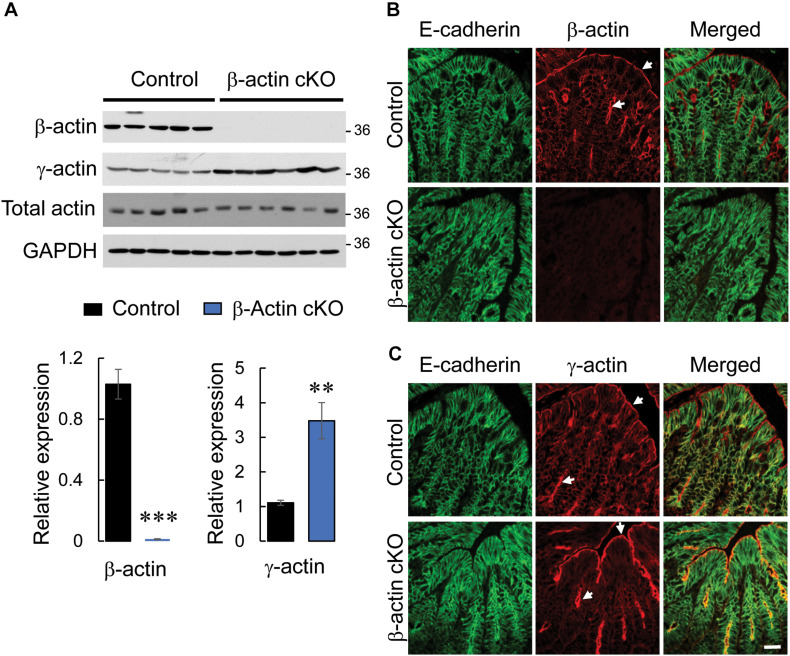
Intestinal epithelial-specific knockout of β-actin in mice results in a compensatory increase in γ-actin expression. **(A)** Immunoblotting analysis of the expression of cytoplasmic actin isoforms (β-actin and γ-actin) and total actin in colonic epithelial scrapings obtained from control and β-actin cKO mice. Representative immunoblots and densitometric quantification of β-actin and γ-actin expression are shown. Data is presented as a mean ± SE (*n* = 5); ^∗∗^*p* < 0.005, ^∗∗∗^*p* < 0.0005. **(B,C)** Dual immunofluorescence labeling of either β-actin **(B)** or γ-actin **(C)** (red) and E-cadherin (green) in full-thickness colonic tissue sections obtained from control and β-actin cKO mice. Arrows indicate the predominant accumulation of both cytoplasmic actin isoforms at the apical pole of colonic epithelial cells. Scale bar, 20 μm.

β-actin is known to be essential for the early stages of development and its total knockout results in embryonic lethality (Shawlot et al., 1998; Bunnell et al., 2011). In order to study the physiological functions of this cytoskeletal protein in the gastrointestinal tract, we generated a mouse model with intestinal epithelium-specific knockout of β-actin. β-actin floxed mice were crossed with mice that express a Cre recombinase driven by a constitutively-active villin promoter. Immunoblotting analysis and immunofluorescence labeling were used to demonstrate the high efficiency and specificity of β-actin knockout in the intestinal epithelium. Expression of β-actin protein was undetectable in colonic epithelial cell scrapings collected from β-actin flox/villin Cre homozygous (referred hereafter as β-actin cKO) mice (Figure 1A). Furthermore, according to immunofluorescence labeling of whole thickness colonic sections, the targeted protein was markedly depleted in E-cadherin-positive colonic epithelial cells (Figure 1B), but was abundantly expressed in non-epithelial compartments, such as the *lamina propria* and *muscularis propria* (Supplementary Figure 1, arrows). Knockout of β-actin resulted in a more than threefold upregulation of γ-actin level (Figure 1A), but did not induce α-smooth muscle actin protein expression in colonic epithelial scrapings (data not shown). Since β-actin and γ-actin have similar cellular localization in the intestinal mucosa of control mice (Figures 1B,C, arrows), the increased accumulation of apical and junction-associated γ-actin (Figure 1C) could serve as a compensatory response to the loss of β-actin expression. Consistent with such compensatory mechanism, F-actin labeling with a fluorescent phalloidin probe did not display significant alterations of the actin cytoskeletal architecture in β-actin-deficient intestinal epithelium (Supplementary Figure 2, arrows). Upregulation of γ-actin can explain the unchanged level of total actin in β-actin-depleted epithelial cells (Figure 1A). This important observation rules out the possibility that the altered gastrointestinal responses of β-actin cKO animals, described below, reflect the non-specific consequences of decreased actin concentration in intestinal epithelial cells. β-actin cKO mice were born healthy and did not show growth retardation or symptoms of gastrointestinal inflammation, such as spontaneous diarrhea, rectal prolapses, or bleeding (data not shown). Furthermore, intestinal epithelium-specific knockout of β-actin did not cause noticeable morphological abnormalities to the colonic and ileal mucosa (Supplementary Figure 3).

**FIGURE 2:**
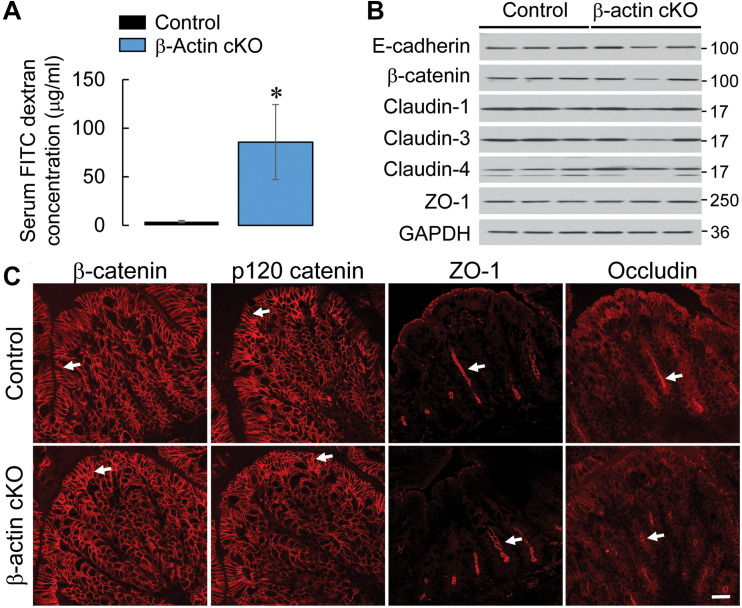
Intestinal epithelial-specific knockout of β-actin increases intestinal permeability without causing significant alterations in the structure and composition of epithelial junctions. **(A)** Intestinal permeability of control and β-actin cKO mice *in vivo* was determined by measuring gut-to-blood passage of FITC-dextran. Data is presented as a mean ± SE (*n* = 5); ^∗^*p* < 0.05. **(B)** Immunoblotting analysis of the expression of selected AJ and TJ proteins in colonic epithelial scrapings obtained from control and β-actin cKO mice. **(C)** Immunofluorescence labeling and confocal microscopy of AJ (β-catenin and p120 catenin) and TJ (ZO-1, occludin) in colonic sections obtained from control and β-actin cKO mice. Arrows indicate similar localization of junctional proteins in β-actin cKO animals and their control littermates. Scale bar, 20 μm.

Given our previous findings, that down-regulation of β-actin expression disrupts the integrity of intestinal epithelial barrier *in vitro* (Baranwal et al., 2012), we investigated the effects of β-actin knockout on gut barrier permeability *in vivo*. Remarkably, healthy β-actin cKO mice demonstrated an approximately 20-fold increase in the transmucosal flux of FITC-dextran as compared to control animals (Figure 2A). The observed leakiness of the β-actin-depleted epithelial barrier was not accompanied by altered expression of key AJ and TJ proteins (Figure 2B). Furthermore, immunofluorescence labeling did not show distinct changes in junctional localization of E-cadherin, β-catenin, p120-catenin, ZO-1, and occludin in the colonic mucosa of β-actin cKO mice (Figures 1B,C, 2C, arrows). This data suggests that the increase in gut permeability caused by depletion of intestinal epithelial β-actin *in vivo* is not mediated by the abnormal assembly of epithelial apical junctions.

### Intestinal Epithelial Specific Knockout of β-Actin Exaggerates the Severity of Experimental Colitis

**FIGURE 3:**
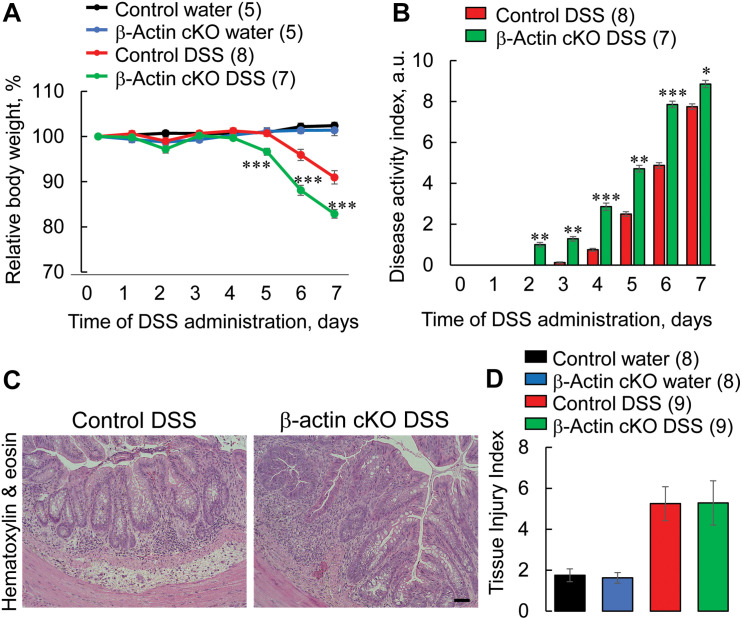
Intestinal epithelial-specific knockout of β-actin exacerbates disease severity during DSS-induced colitis. Control and β-actin cKO mice were exposed to 3% DSS in drinking water, or regular drinking water as a control, for 7 days. **(A)** Body weight loss and **(B)** the disease activity index were calculated for the duration of DSS treatment. Distal colonic sections of β-actin cKO mice and their controls were fixed, paraffin embedded, and stained with hematoxylin & eosin. Representative images **(C)** and a calculated tissue injury index **(D)** are shown. Number of animals of each group is shown in parentheses. Data is presented as a mean ± SE, ^∗^*p* < 0.05, ^∗∗^*p* < 0.005, ^∗∗∗^*p* < 0.0005. Scale bar, 100 μm.

Increased permeability of the epithelial barrier could result in an exaggerated and prolonged inflammatory response in the gut (Marchiando et al., 2010; Lee et al., 2018; Fasano, 2020). Thus, we next investigated whether destabilized gut barrier in β-actin cKO mice affects the development of mucosal injury and inflammation using a dextran sodium sulfate (DSS) model of acute colitis. DSS administration caused more severe intestinal disease in β-actin cKO mice, as compared to control littermates (Figure 3). This effect was revealed in their significantly higher body weight loss (Figure 3A), and a higher disease activity index (Figure 3B). Despite having more severe symptoms of gastrointestinal disorder, β-actin cKO mice did not show more pronounced DSS-induced abnormalities of the colonic mucosa, according to examination of hematoxylin & eosin (H&E)-stained whole thickness sections of distal colon. The cumulative tissue injury index, which was calculated based on the extent of mucosal inflammation, leukocyte infiltration, submucosal fibrosis and epithelial erosion, was not significantly different in DSS-treated β-actin cKO mice compared to their control littermates (Figures 3C,D).

### Intestinal Epithelial Specific Knockout of β-Actin Exacerbates the Inflammatory Response in Colonic Mucosa

**FIGURE 4:**
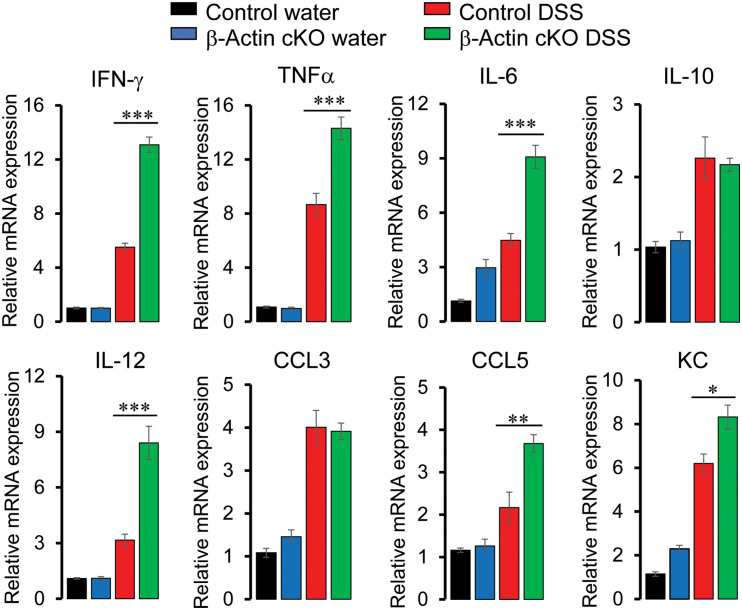
Intestinal epithelial-specific knockout of β-actin increases colonic expression of proinflammatory cytokines and chemokines during DSS colitis. Control and β-actin cKO mice were exposed to 3% DSS in drinking water, or regular water as a control, for 7 days. mRNA was isolated from the collected colonic samples. Real-time quantitative RT-PCR was used to analyze the expression of different cytokines and chemokines. Data is presented as mean ± SE (*n* = 5), ^∗^*p* < 0.05, ^∗∗^*p* < 0.01, ^∗∗∗^*p* < 0.005.

Since histochemical evaluation of H&E-stained tissue sections provides just a general snapshot of the mucosal architecture, we used more specific and sensitive assays to evaluate the different aspects of tissue inflammation and injury in DSS-treated animals. A quantitative RT-PCR analysis was utilized to examine the expression of different proinflammatory cytokines and chemokines in whole thickness colonic samples on day 7 of DSS administration. Expectedly, mRNA expression of the tested cytokines [TNFα, interferon (IFN)-γ, interleukins (IL) 6, 10, and 12], and chemokines [chemokine ligands (CCL) 3 and 5, keratinocyte-derived chemokine (KC)] was upregulated by DSS treatment of the murine colon (Figure 4). Interestingly, expression of IFN-γ, TNFα, IL-6, IL-12, CCL5, and KC was significantly higher in tissue samples of DSS-treated β-actin cKO mice, as compared to their control littermates (Figure 4). It is noteworthy that mRNA expression of the examined cytokines and chemokines was not elevated in colonic tissue of normal β-actin cKO mice lacking DSS treatment (Figure 4). This indicates that leaky gut barrier in the knockout animals does not lead to the development of spontaneous mucosal inflammation.

**FIGURE 5:**
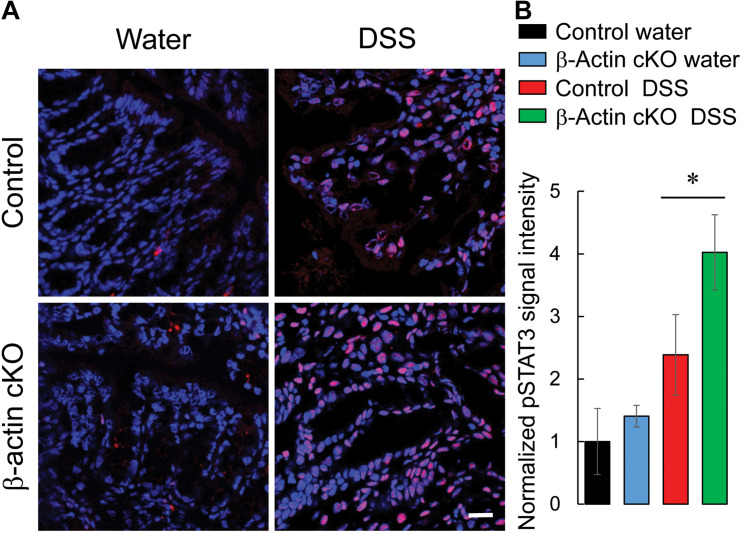
Intestinal epithelial-specific knockout of β-cytoplasmic actin increases the activation of STAT3 in colonic mucosa during DSS colitis. Control and β-actin cKO mice were exposed to 3% DSS in drinking water, or regular water, for 7 days. Harvested colonic samples were immunofluorescently labeled for phospho-(p)-STAT3 (red) and counter-labeled with nuclear stain, DAPI (blue). Representative images **(A)** and quantification of pSTAT3 labeling **(B)** are shown. Data is presented as a mean ± SE (*n* = 4), *p < 0.05. Scale bar, 20 μm.

Next we sought to investigate if increased cytokine expression leads to the exaggerated inflammatory signaling in the colonic mucosa of DSS-exposed β-actin cKO mice. Expression of active (phosphorylated) STAT3 was used as a biochemical readout for inflammatory signaling events, since STAT3 is a common signaling molecule activated by multiple cytokine receptors (Ahmad et al., 2014; Serrano et al., 2019). Colonic tissue sections were immunofluorescently labeled for phospho-STAT3 to visualize STAT3 activation. The level of activated STAT3 was markedly increased with DSS treatment, and such increase was significantly higher in the colonic tissue of β-actin cKO mice, compared to their control littermates (Figure 5).

**FIGURE 6:**
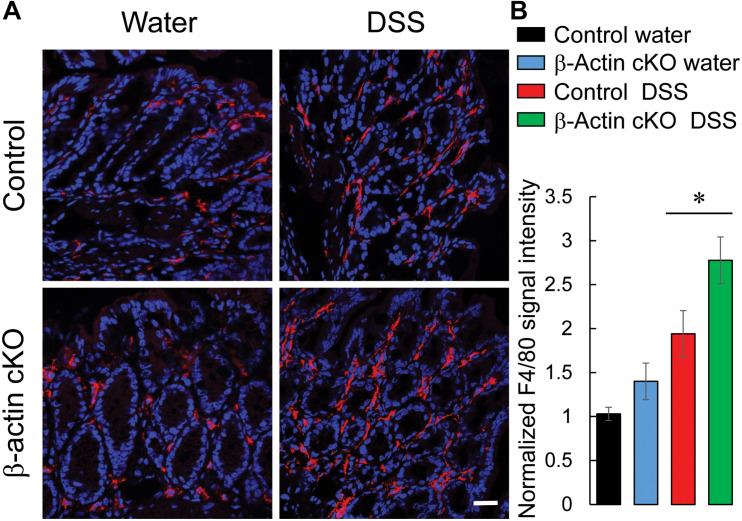
Loss of intestinal epithelial β-actin increases the accumulation of macrophages in inflamed colonic mucosa. Control and β-actin cKO mice were exposed to 3% DSS in drinking water, or regular water, for 7 days. Harvested colonic samples were immunofluorescently labeled for a specific macrophage marker, F4/80 (red), and counter-labeled with nuclear stain, DAPI (blue). Representative images **(A)** and quantification of F4/80 labeling **(B)** are shown. Data is presented as a mean ± SE (*n* = 6), ^∗^*p* < 0.05. Scale bar, 20 μm.

We also examined whether loss of β-actin expression exaggerates intestinal inflammation by enhancing the accumulation of different classes of leukocytes in the colonic mucosa. Immunofluorescence labeling of F4/80, myeloperoxidase (MPO) and CD4 antigens was utilized to detect macrophages, neutrophils, and T lymphocytes, respectively. DSS administration upregulated the amounts of all three types of leukocytes in mouse colonic tissue (Figure 6 and Supplementary Figure 4). Interestingly, the number of macrophages was significantly higher in the distal colon of DSS-treated β-actin KO mice, compared to control animals (Figure 6). Conversely, no significant differences in the colonic recruitment of neutrophils and T cells was observed between knockout and control mice (Supplementary Figure 4). Taken together, our data strongly suggests that depletion of intestinal epithelial β-actin exaggerates mucosal inflammation during acute experimental colitis *in vivo*.

### Intestinal Epithelial Specific Knockout of β-Actin Enhances Inflammation-Induced Cell Death

**FIGURE 7:**
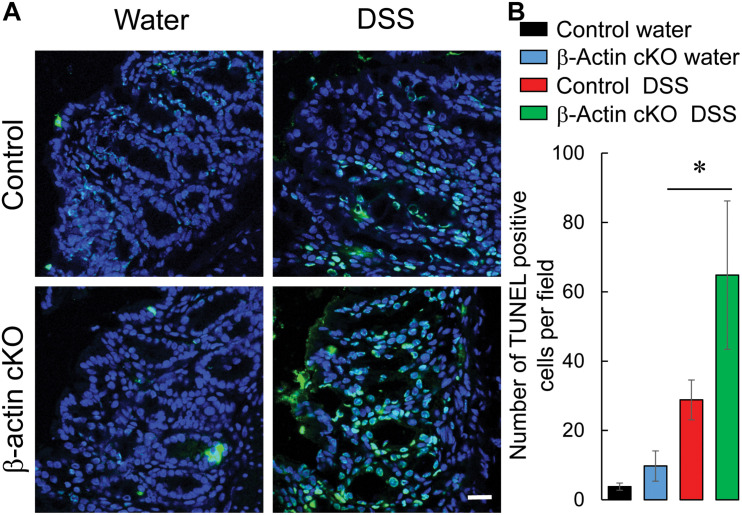
Intestinal epithelial-specific knockout of β-actin increases cell death in intestinal mucosa during DSS colitis. Colonic sections of DSS and water-exposed control, and β-actin cKO, mice were subjected to TUNEL labeling (green) to visualize dead cells and counter-labeled with nuclear stain, DAPI (blue). Representative images **(A)** and quantification of TUNEL labeling **(B)** are shown. Data is presented as a mean ± SE (*n* = 5), ^∗^*p* < 0.05. Scale bar, 20 μm.

**FIGURE 8:**
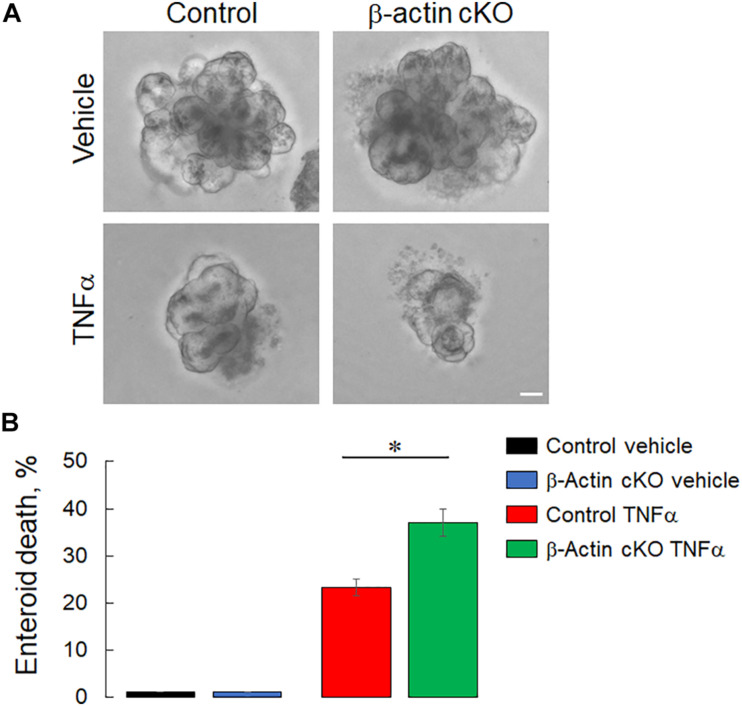
Loss of intestinal epithelial β-actin increases TNFα-induced cell death *ex vivo*. Small intestinal crypts were isolated from β-actin cKO and control mice and embedded into Matrigel to generate intestinal enteroids. Differentiated enteroids were treated for 12 h with murine TNFα (100 ng/ml) and the number of dead enteroids, as distinguished by their morphology, was counted. Representative bright field images **(A)** and quantification of enteroid survival after TNFα treatment **(B)** are shown. Data is presented as a mean ± SE (*n* = 3), ^∗^*p* < 0.05. Scale bar, 50 μm.

Mucosal injury during DSS colitis is known to develop due to excessive cell death caused by either direct toxic effects of DSS, or by endogenously produced inflammatory mediators (Ahmad et al., 2014; Naydenov et al., 2016). Therefore, we asked whether the more severe colitis state observed in β-actin cKO mice is associated with higher intestinal epithelial cell death. Because of its broad ability to detect both apoptotic and non-apoptotic types of cell death, a TUNEL assay was utilized (Fink and Cookson, 2005). DSS treatment caused a marked increase in TUNEL labeling of murine colonic sections, indicating increased cell death (Figure 7). The magnitude of this cell death was significantly higher in DSS-treated β-actin cKO mice, compared to their controls (Figure 7). Finally, we sought to investigate if the increased cell death in β-actin-deficient intestinal mucosa reflects increased sensitivity to the direct toxicity of DSS or is due to the exaggerated response to cell-death inducing inflammatory mediators. Enteroids derived from ileal crypts of control and β-actin cKO mice were cultured *ex vivo* in Matrigel and were exposed to known apoptosis-inducing cytokine TNFα. Non-stimulated β-actin-deficient enteroids did not show impaired growth and differentiation (budding), compared to enteroids derived from control animals (Figure 8). However, the loss of β-actin significantly increased the susceptibility of enteroids to TNFα-induced cell death (Figure 8). Taken together, this data indicates that β-actin plays protective roles in the intestinal mucosa by attenuating inflammation-induced epithelial cell death.

## Discussion

Intestinal epithelial cells express two nearly identical actin isoforms, β-actin and γ-actin, which play non-redundant roles in regulating the assembly of different apical junctional complexes and the early stages of intestinal epithelial morphogenesis *in vitro* (Baranwal et al., 2012; Gutierrez et al., 2014; Cruz et al., 2015). The present study is the first to investigate the physiological functions of β-actin in the intestinal epithelium *in vivo*. We report that the specific loss of this cytoplasmic actin isoform in murine intestinal epithelium results in subtle physiological changes that include increased epithelial permeability, without causing gross abnormalities in intestinal architecture or development. Such β-actin knockout is accompanied by significant upregulation of γ-actin protein expression (Figure 1), strongly suggesting that γ-actin could functionally compensate for the loss of β-actin in normal intestinal epithelium *in vivo*. The notion of functional compensation among actin isoforms is in line with published studies that examined the effects of conditional knockout of β-actin in different tissues. For example, conditional knockout of β-actin in hair cells did not cause defects in stereocilia formation or auditory function in mice due to compensation from γ-actin (Perrin et al., 2010). Furthermore, mature adult mice with muscle-specific knockout of β-actin did not show major defects in glucose tolerance and insulin stimulated glucose transport into muscle (Madsen et al., 2018). Finally, deletion of β-actin in the central nervous system resulted in limited abnormalities of tissue architecture, localized to the hippocampus and cerebellum (Cheever et al., 2012).

Although intestinal epithelial specific knockout of β-actin increases the permeability of the gut barrier, this barrier defect is caused neither by altered structure or composition of tight or adherens junctions, nor by gross abnormalities in the apical F-actin cytoskeleton (Figure 2 and Supplementary Figure 2). It is likely that the observed increase in barrier permeability reflects the altered balance of β-actin and γ-actin in the junction-associated cytoskeleton. Actin isoform-specific changes in filament turnover and interactions with actin binding proteins have been previously reported *in vitro* (Bergeron et al., 2010; Baranwal et al., 2012; Muller et al., 2013; Lechuga et al., 2014; Chen et al., 2017). Thus, in a cell-free assay, γ-actin displays a slower polymerization rate and forms more stable filaments as compared to β-actin (Bergeron et al., 2010). Furthermore, β-actin selectively interacts with important regulators of actin filament polymerization, such as diaphanous-related formin 3 and beta-cap73 (Shuster et al., 1996; Chen et al., 2017). On the other hand, γ-actin was shown to specifically bind the Arp2/3 complex and a key actin filament depolymerizing protein, cofilin-1 (Dugina et al., 2015). One could therefore suggest that barrier leakiness in β-actin deficient intestinal epithelium is mediated by subtle changes to the spatial organization, turnover rate, or contractility of γ-actin-enriched junction-associated F-actin bundles.

The normal morphology of apical junctions in β-actin-deficient intestinal epithelium *in vivo* contradicts our previous *in vitro* finding that loss of this actin isoform disrupts AJ assembly and the apico-basal cell polarity in cultured colonic epithelial cell monolayers (Baranwal et al., 2012). However, accumulating evidence suggests that genetic perturbations of the actin cytoskeleton have much milder effects on intestinal epithelial homeostasis and gut barrier permeability in mice, as compared to their effects in model intestinal cell monolayers *in vitro*. For example, Arp2/3 dependent actin polymerization was implicated in the formation of epithelial junctions in cultured colonic epithelial cells (Ivanov et al., 2005) but was superfluous to the establishment of normal epithelial junctions and cell polarity in the intestinal epithelium *in vivo* (Zhou et al., 2015). Furthermore, mice with total knockout of the actin depolymerizing factor did not display leakiness of the gut barrier, though knockdown of this protein in cultured colonic epithelial cell monolayers increased epithelial permeability (Wang et al., 2016). These differing responses could be explained by the different levels of mechanical stress applied to epithelial junctions *in vitro* and *in vivo*. Specifically, epithelial cell monolayers cultured on either glass coverslips or plastic membrane filters are adapted to high tensile forces due to their attachment to stiff substrates. Since the mechanical forces transduced by the actomyosin cytoskeleton are key regulators of the assembly and permeability of apical junctions (Mege and Ishiyama, 2017; Sluysmans et al., 2017; Charras and Yap, 2018; Varadarajan et al., 2019), even modest perturbation of actin cytoskeletal tension and contractility could result in substantial changes in junctional architecture. Contrastingly, the intestinal epithelial barrier develops in a much softer tissue environment *in vivo* with weaker mechanical forces applied to epithelial junctions. As a result, epithelial junctions *in vivo* could be less responsive to alterations in the weaker mechanical forces caused by perturbed organization of the actin cytoskeleton.

Our study demonstrates that increased gut barrier permeability in β-actin cKO mice does not result in the development of spontaneous mucosal inflammation. This finding is in keeping with prior studies that document the absence of spontaneous colitis in other mouse models characterizing by leaky gut barrier. These models include mice with total knockout of either JAM-A (Laukoetter et al., 2007), or cortactin (Citalan-Madrid et al., 2017), as well as mice with intestinal epithelial specific loss of non-muscle myosin (NM) IIA (Naydenov et al., 2016). Some compensatory mechanisms should exist to prevent gut pathogens from taking advantage of destabilized gut barrier, thus invading the intestinal mucosa. While such mechanisms have not been investigated in β-actin cKO mice, they could be similar to mechanisms reported in other knockout animals with increased gut permeability. For example, an immune protective mechanism that prevents spontaneous gastrointestinal disease has been previously described in JAM-A knockout mice (Khounlotham et al., 2012). This mechanism involves the production of TGF-β by T cells, which in turn stimulates the production of antibacterial IgA. Since similar upregulation of this TGF-β/IgA pathway has been observed in the colonic tissue of mice with intestinal epithelial knockout of NM IIA (Naydenov et al., 2016), such adaptive immune response may represent a common mucosal protective mechanism within the context of increased gut permeability.

Our study shows for the first time that intestinal epithelial β-actin plays a protective role during mucosal inflammation *in vivo*. Indeed β-actin cKO mice display an exaggerated pathophysiological manifestation of experimental colitis (Figure 3) along with more pronounced inflammatory signaling and tissue injury responses (Figures 4–7). Several mechanisms can mediate the increased production of inflammatory cytokines and chemokines in the intestinal tissue of β-actin cKO mice. One such mechanism involves leaky epithelial barrier in these animals, facilitating the influx of luminal pathogens and leading to a stronger activation of the mucosal immune system. A complementary mechanism may involve the direct modulation of inflammatory mediator expression in intestinal epithelial cells caused by a dramatic shift in their β-actin/γ-actins balance. Indeed, actin is an important regulator of the transcriptional program in different cells and tissues (Miyamoto and Gurdon, 2013; Percipalle and Vartiainen, 2019), and cytoplasmic actin-specific effects on gene expression are well-documented (Bunnell et al., 2011; Tondeleir et al., 2012; Lechuga et al., 2014).

The observed exaggeration of DSS colitis in β-actin cKO mice concurs with previous studies showing that perturbation of either actin filament turnover (Wang et al., 2016; Citalan-Madrid et al., 2017) or actomyosin contractility in the intestinal epithelium (Su et al., 2009; Naydenov et al., 2016) exaggerates the severity of intestinal inflammation *in vivo*. These findings may also have significant clinical relevance. The increased permeability of the gut barrier is a well-recognized feature of different gastrointestinal and systemic inflammatory disorders (Marchiando et al., 2010; Lee et al., 2018; Fasano, 2020). However, whether or not such leaky gut accelerates mucosal inflammation, or inhibits it due to immune suppression remains under debate (Ahmad et al., 2017). Since β-actin cKO mice and other mouse models with selective perturbation of the intestinal epithelial cytoskeleton (Su et al., 2009; Naydenov et al., 2016) possess two key common features, a leaky gut barrier in otherwise healthy animals and exaggerated mucosal inflammation during experimental colitis, these models provide strong support for the idea that leaky gut barrier could worsen the severity of gastrointestinal diseases in human patients.

While the exact mechanisms of the exaggerated mucosal inflammation β-actin-depleted intestinal mucosa are yet to be investigated, this response could be at least partially mediated by accelerated cell death. Increased cell death is a known factor promoting mucosal injury and attenuating the repair processes in both animal models of colitis and human IBD patients (Blander, 2018; Thoo et al., 2019). Our data demonstrates a markedly accelerated cell death in the colonic mucosa of DSS-treated β-actin cKO animals (Figure 7). Furthermore, enteroids developed from these knockout mice appear to be highly sensitive to TNFα-induced cytotoxicity *ex vivo* (Figure 8). To the best of our knowledge, this is the first direct data identifying β-actin as a positive regulator of human epithelial cell survival. Previous studies provided only indirect evidence linking β-actin with cell death. For example, decreased β-actin expression was associated with actinomycin D-induced apoptosis in a hematopoietic cell line (Naora and Naora, 1995) and in TNFα-exposed vascular endothelial cells (Kohno et al., 1993). Furthermore, chemotherapy-induced apoptosis of leukemic cells was paralleled by the selective phosphorylation and depolymerization of β-actin filaments (Wang et al., 2008). Finally, a cell permeable β-actin-targeting peptide was shown to trigger the death of several human cancer cell lines (Arruda et al., 2012). Given the established ability of β-actin filaments to regulate crucial signaling cascades in different cellular compartments, it would be important to determine the exact molecular events that mediate the described prosurvival activity of β-actin in epithelial cells.

## Data Availability Statement

The raw data supporting the conclusions of this article will be made available by the authors, without undue reservation, to any qualified researcher.

## Ethics Statement

The animal study was reviewed and approved by the Virginia Commonwealth University and Lerner Research Institute Animal Care and Use Committees in accordance with the National Institutes of Health Animal Care and Use Guidelines.

## Author Contributions

SL and NN performed experiments, analyzed and interpreted data, and prepared figures. AF performed experiments and participated in data analysis. JE provided Actb1 floxed animals and helped with manuscript preparation. MC was involved in the design, data analysis and interpretation for the experimental colitis study. AI conceived the study, supervised the project and wrote the manuscript. All authors have read and approved the manuscript.

## Conflict of Interest

The authors declare that the research was conducted in the absence of any commercial or financial relationships that could be construed as a potential conflict of interest.

## Supplementary Material

Supplementary Figure 1 Selective depletion of β-actin in the intestinal epithelium of β-actin cKO mice. Whole thickness colonic sections of control and β-actin cKO mice were dual immunofluorescently labeled for β-actin (red) and E-cadherin (green). Confocal microscopy images show selective loss of β-actin labeling in E-cadherin-positive epithelial cells and abundant expression of this protein in subepithelial colonic compartments of β-actin cKO mice (arrows). Scale bar, 20 μm.

Supplementary Figure 2 Intestinal epithelial-specific knockout of β-actin does not affect organization of the epithelial actin cytoskeleton. Fluorescence labeling of F-actin in colonic tissue sections obtained from control and β-actin cKO mice. Arrows indicate normal architecture of epithelial F-actin at the colonic surface and in the crypts. Scale bar, 20 μm.

Supplementary Figure 3 Intestinal epithelial-specific knockout of β-actin does not affect normal architecture of the intestinal mucosa. Normal architecture of colonic epithelium, as shown by H&E staining in the colonic and ileal sections of wild-type and β-actin cKO mice. Scale bar, 100 μm.

Supplementary Figure 4 Loss of intestinal epithelial β-actin does not affect T lymphocyte and neutrophil infiltration in inflamed colonic mucosa. Control and β-actin cKO mice were exposed to 3% DSS in drinking water, or regular water for 7 days. Whole thickness colonic sections were immunofluorescently labeled for either a specific neutrophil marker, myeloperoxidase (MPO, **A,B**), or a specific T cell marker, CD4 **(C,D)** and counter-labeled with stained nuclei (blue). Representative images **(A,C)** and quantification of the immunolabeling **(B,D)** are shown. Data is presented as mean ± SE (*n* = 5). Scale bar, 20 μm.

## Abbreviations

AJ: adherens junctions
CCL: chemokine ligand
cKO: conditional knockout
DSS: dextran sulfate sodium
FITC: fluorescein isothiocyanate
H&E: hematoxylin and eosin
IBD: inflammatory bowel diseases
IFNγ: interferon γ
IL: interleukin
JAM-A: junctional adhesion molecule-A
MPO: myeloperoxidase
NM II: non-muscle myosin II
TJ: tight junctions
TNFα: tumor necrosis factor alpha
TUNEL: terminal deoxynucleotidyl transferase dUTP nick end labeling
ZO: zonula occludens.
