# Supplementary material for: Loss of β-Cytoplasmic Actin in the Intestinal Epithelium Increases Gut Barrier Permeability in vivo and Exaggerates the Severity of Experimental Colitis
Source: Front Cell Dev Biol. 2020 Oct 23;8:588836. doi: 10.3389/fcell.2020.588836 (PMC7644907; doi:10.3389/fcell.2020.588836)
Supplement: Supplementary file 1 [file Table_1.DOCX]

**Supplemental Table:** List of antibodies used for immunofluorescence labeling (IF) and immunoblotting (IB) analysis.

| **Antibody** | **Source** | **Cat. #** | **Company** | **Application and dilution** |
| --- | --- | --- | --- | --- |
| β-actin | Mouse | NA | Dugina V et al., (2009) J Cell Sci 122:2980-88 | IF (1:200), IB (1:1000) |
| γ-actin | Mouse | NA | Dugina V et al., (2009) J Cell Sci 122:2980-88 | IF (1:200), IB (1:3000) |
| Total actin | Mouse | MAB1501 | EMD Millipore | IB (1:5000) |
| Occludin | Rabbit | 711500 | ThermoFisher Scientific | IF (1:200), IB (1:1000) |
| ZO-1 | Rabbit | 40-2200 | ThermoFisher Scientific | IF (1:200), IB (1:1000) |
| Claudin-1 | Rabbit | 187362 | ThermoFisher Scientific | IB (1:1000) |
| Claudin-3 | Rabbit | 34-1700 | ThermoFisher Scientific | IB (1:1000) |
| Claudin-4 | Mouse | 32-9400 | ThermoFisher Scientific | IB (1:1000) |
| β-catenin | Mouse | 610154 | BD Biosciences | IB (1:1000) |
| β-catenin | Rabbit | C2206 | Sigma-Aldrich | IF (1:200) |
| p120-catenin | Mouse | 610133 | BD Biosciences | IF (1:200), IB (1:1000) |
| E-cadherin | Mouse | 610181 | BD Biosciences | IB (1:1000) |
| E-cadherin | Goat | AF748 | R&D System | IF (1:500) |
| CD4 | Mouse | 550278 | BD Biosciences | IF (1:200) |
| GAPDH | Rabbit | 2118 | Cell Signaling | IB (1:10000) |
| phospho-STAT3 | Rabbit | 9131 | Cell Signaling | IF (1:200) |
| F4/80 | Rat | MCA497 | Bio-Rad Laboratories | IF (1:200) |
| MPO | Rabbit | ab9535 | Abcam | IF (1:200) |
