# Supplementary figures and images for: Loss of β-Cytoplasmic Actin in the Intestinal Epithelium Increases Gut Barrier Permeability in vivo and Exaggerates the Severity of Experimental Colitis
Source: Front Cell Dev Biol. 2020 Oct 23;8:588836. doi: 10.3389/fcell.2020.588836 (PMC7644907; doi:10.3389/fcell.2020.588836)

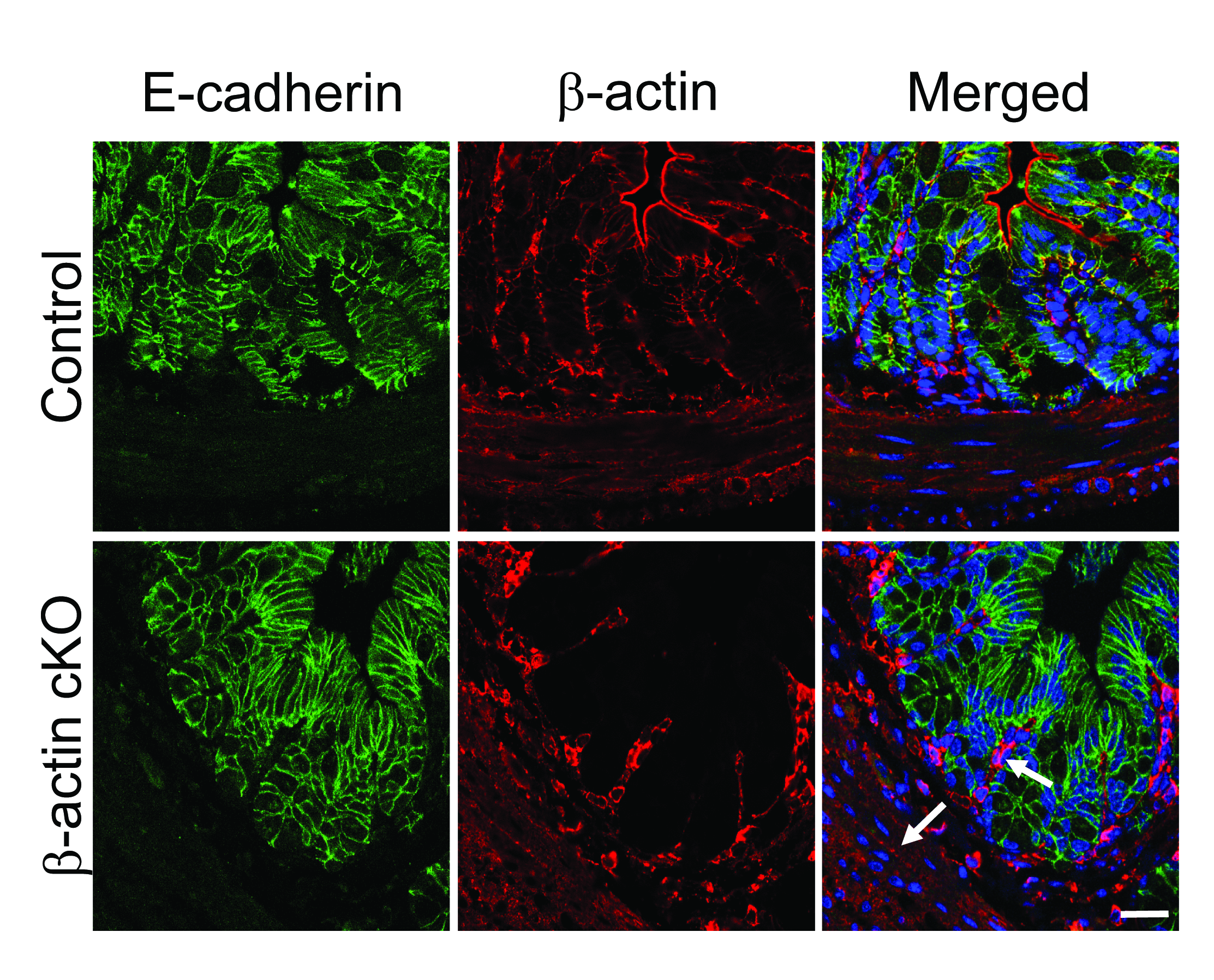

Supplement: Supplementary Figure 1 — Selective depletion of β-actin in the intestinal epithelium of β-actin cKO mice. Whole thickness colonic sections of control and β-actin cKO mice were dual immunofluorescently labeled for β-actin (red) and E-cadherin (green). Confocal microscopy images show selective loss of β-actin labeling in E-cadherin-positive epithelial cells and abundant expression of this protein in subepithelial colonic compartments of β-actin cKO mice (arrows). Scale bar, 20 μm. [file Image_1.TIF]

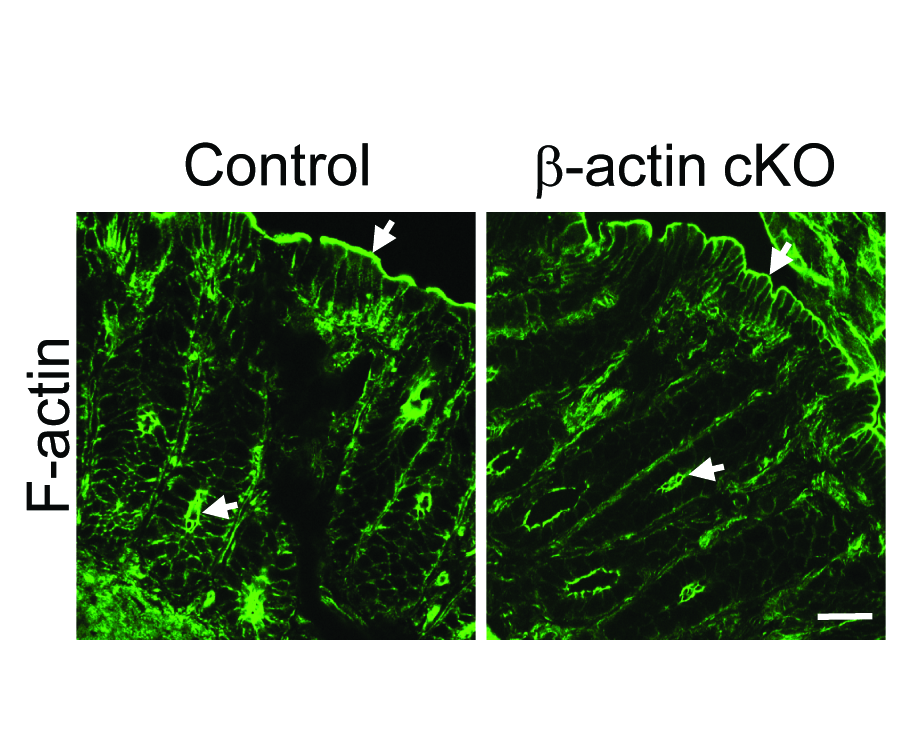

Supplement: Supplementary Figure 2 — Intestinal epithelial-specific knockout of β-actin does not affect organization of the epithelial actin cytoskeleton. Fluorescence labeling of F-actin in colonic tissue sections obtained from control and β-actin cKO mice. Arrows indicate normal architecture of epithelial F-actin at the colonic surface and in the crypts. Scale bar, 20 μm. [file Image_2.TIF]

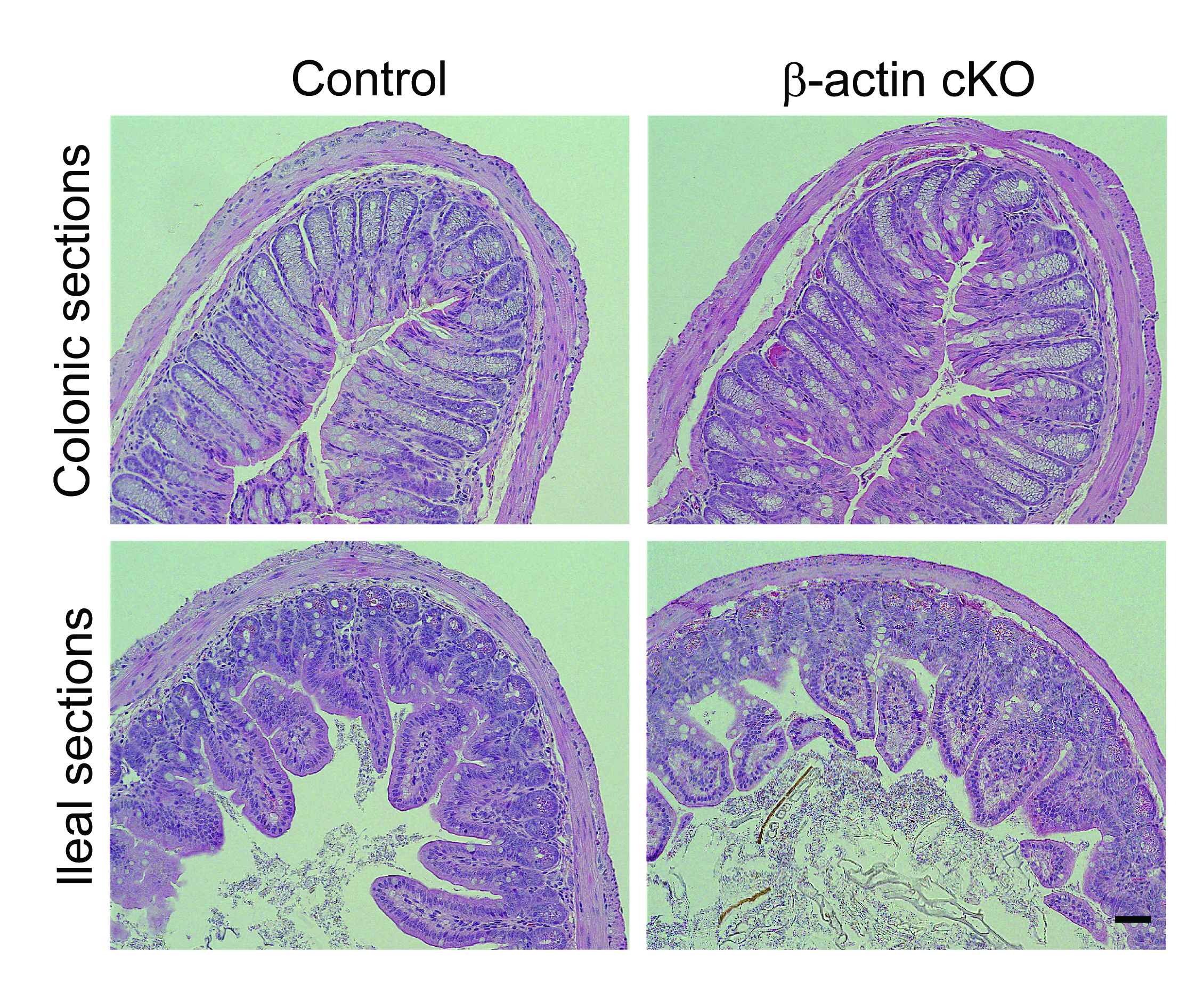

Supplement: Supplementary Figure 3 — Intestinal epithelial-specific knockout of β-actin does not affect normal architecture of the intestinal mucosa. Normal architecture of colonic epithelium, as shown by H&E staining in the colonic and ileal sections of wild-type and β-actin cKO mice. Scale bar, 100 μm. [file Image_3.TIF]

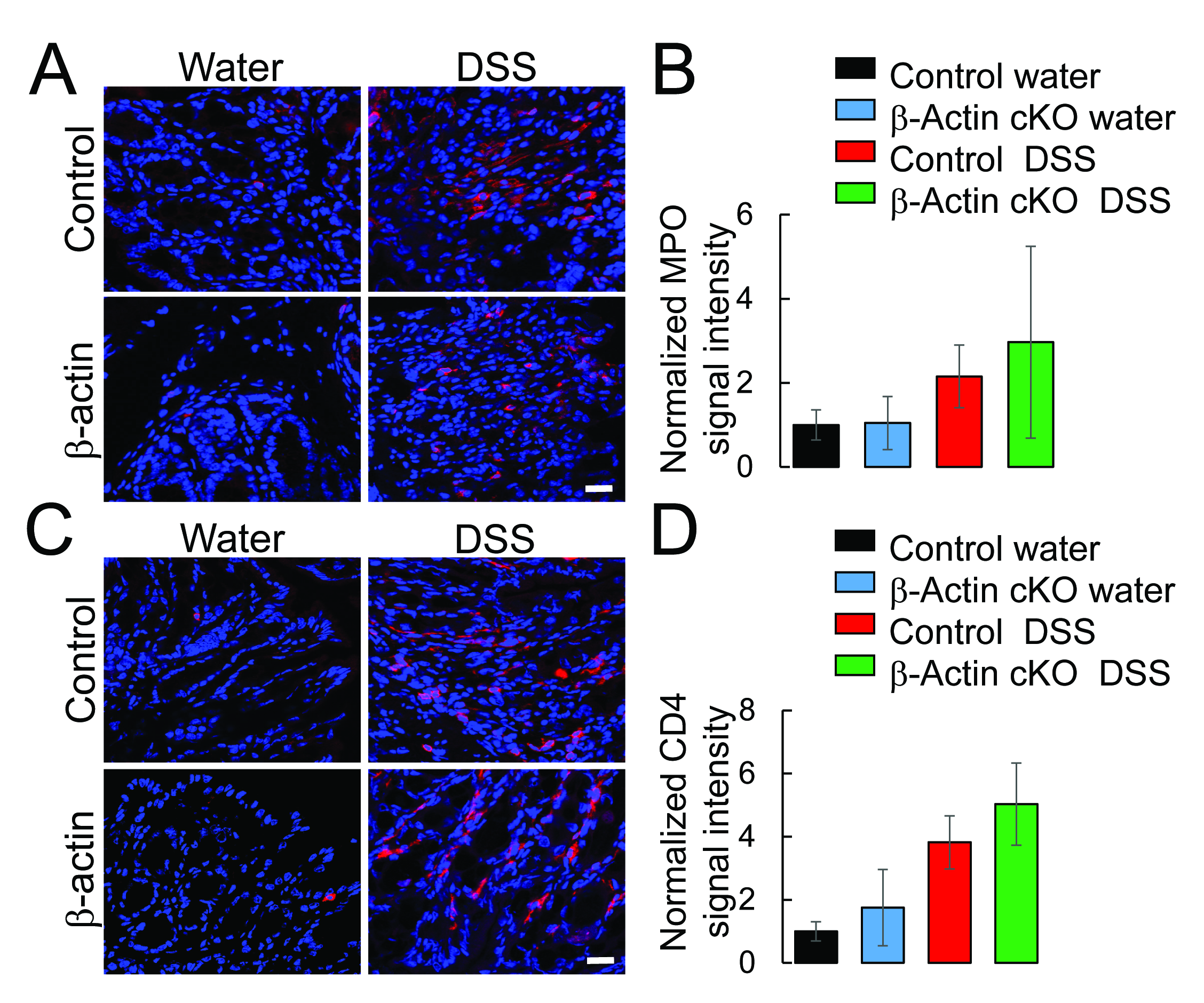

Supplement: Supplementary Figure 4 — Loss of intestinal epithelial β-actin does not affect T lymphocyte and neutrophil infiltration in inflamed colonic mucosa. Control and β-actin cKO mice were exposed to 3% DSS in drinking water, or regular water for 7 days. Whole thickness colonic sections were immunofluorescently labeled for either a specific neutrophil marker, myeloperoxidase (MPO, A,B), or a specific T cell marker, CD4 (C,D) and counter-labeled with stained nuclei (blue). Representative images (A,C) and quantification of the immunolabeling (B,D) are shown. Data is presented as mean ± SE (n = 5). Scale bar, 20 μm. [file Image_4.TIF]
